# Eltoprazine modulated gamma oscillations on ameliorating L‐dopa‐induced dyskinesia in rats

**DOI:** 10.1111/cns.14241

**Published:** 2023-04-30

**Authors:** Yuewei Bi, Pengfei Wang, Jianshen Yu, Zhuyong Wang, Hanjie Yang, Yuhao Deng, Jianwei Guan, Wangming Zhang

**Affiliations:** ^1^ Neurosurgery Center, Department of Pediatric Neurosurgery, Guangdong Provincial Key Laboratory on Brain Function Repair and Regeneration, Zhujiang Hospital Southern Medical University Guangzhou China

**Keywords:** 5‐HT1A receptor, abnormal involuntary movements, electrophysiology, local field potential, PAC, Parkinson's disease, serotonin

## Abstract

**Aim:**

Parkinson's disease (PD) is a pervasive neurodegenerative disease, and levodopa (L‐dopa) is its preferred treatment. The pathophysiological mechanism of levodopa‐induced dyskinesia (LID), the most common complication of long‐term L‐dopa administration, remains obscure. Accumulated evidence suggests that the dopaminergic as well as non‐dopaminergic systems contribute to LID development. As a *5*‐*hydroxytryptamine* 1A/1B receptor agonist, eltoprazine ameliorates dyskinesia, although little is known about its electrophysiological mechanism. The aim of this study was to investigate the cumulative effects of chronic L‐dopa administration and the potential mechanism of eltoprazine's amelioration of dyskinesia at the electrophysiological level in rats.

**Methods:**

Neural electrophysiological analysis techniques were conducted on the acquired local field potential (LFP) data from primary motor cortex (M1) and dorsolateral striatum (DLS) during different pathological states to obtain the information of power spectrum density, theta‐gamma phase–amplitude coupling (PAC), and functional connectivity. Behavior tests and AIMs scoring were performed to verify PD model establishment and evaluate LID severity.

**Results:**

We detected exaggerated gamma activities in the dyskinetic state, with different features and impacts in distinct regions. Gamma oscillations in M1 were narrowband manner, whereas that in DLS had a broadband appearance. Striatal exaggerated theta‐gamma PAC in the LID state contributed to broadband gamma oscillation, and aperiodic‐corrected cortical beta power correlated robustly with aperiodic‐corrected gamma power in M1. M1–DLS coherence and phase‐locking values (PLVs) in the gamma band were enhanced following L‐dopa administration. Eltoprazine intervention reduced gamma oscillations, theta–gamma PAC in the DLS, and coherence and PLVs in the gamma band to alleviate dyskinesia.

**Conclusion:**

Excessive cortical gamma oscillation is a compelling clinical indicator of dyskinesia. The detection of enhanced PAC and functional connectivity of gamma‐band oscillation can be used to guide and optimize deep brain stimulation parameters. Eltoprazine has potential clinical application for dyskinesia.

## Graphical abstract

The cumulative effects of chronic L‐dopa administration on gamma oscillations; The impactors affect gamma oscillations in distinct regions in LID state; The abnormal functional connectivity in LID state; The potential mechanism of eltoprazine's amelioration of dyskinesia at the electrophysiological level in rats.

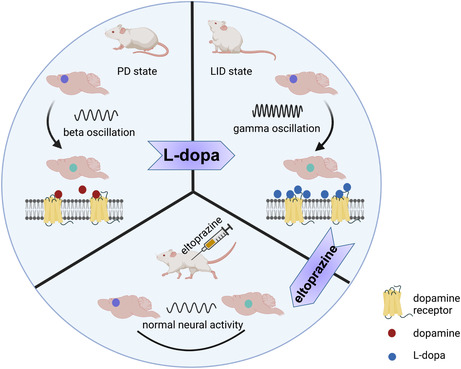

## 1 INTRODUCTION

Parkinson's disease (PD), one of the most common neurodegenerative diseases, imposes a great burden on society. The clinical spectrum of PD consists of motor symptoms such as tremors, rigidity, bradykinesia, and non‐motor symptoms.^1^ Pathologically, PD is characterized by the progressive loss of dopaminergic neurons in the substantia nigra pars compacta (SNc) combined with aberrant cortico–basal ganglia–thalamic (CBT) network neural activity.^2^ Dopamine (DA) replacement therapy is the backbone of PD treatment.^3^ Levodopa‐induced dyskinesia (LID) is a major motor complication of chronic levodopa (L‐dopa) treatment, reflecting the current lack of a standard and effective PD treatment.

Neuroelectrophysiological recording and analysis have been increasingly applied to study the pathophysiology of PD and its complications. As an oscillatory electrical activity, local field potential (LFP) reflects the comprehensive potential changes or synchrony electrical activity results from a group of neurons or synaptic structures in a local region.^4^ Enhanced beta‐band (13–35‐Hz) oscillations and information flow in CBT circuits have been shown to have anti‐kinetic functions in parkinsonian subjects,^5^ and dopaminergic treatment suppresses this enhancement and destruct information flow to ameliorate bradykinesia.^6^ Long‐term L‐dopa administration has prokinetic effects, inducing exaggerated gamma‐band (>35‐Hz) activity associated with abnormal involuntary movements.^7^ Recent studies have examined L‐dopa‐induced excessive gamma oscillations in broadly distributed neural networks, such as those of the striatum^8^ and motor cortex.^9^ Basic research has shown that exaggerated narrowband gamma oscillations in the cortex are associated with dyskinesia and may serve as control signals for adaptive deep brain stimulation (DBS).^10^ Clinical electrophysiological data suggest that broadband gamma oscillations occur in the subthalamic nucleus (STN)^11^ and internal globus pallidus^12^ in patients with advanced PD and dyskinesia. However, the functional consequences of enhanced gamma oscillations in the PD and LID states remain incompletely understood.

Phase–amplitude coupling (PAC) is a representation of cross‐frequency coupling in which the low‐frequency phase modulates the high‐frequency amplitude. PAC alterations have been associated with multiple neurological disorders,^4^ such as PD, Alzheimer's disease, schizophrenia, and epilepsy. The relationship between exaggerated beta–gamma PAC in the cortex and motor impairment in patients with PD has been investigated.^13^ Beta‐band power is known to be related to the severity of PD symptoms, but the correlation between beta and gamma power remains obscure. A clinical study demonstrated that phase‐targeted DBS modulated cortical PAC, leading to motor improvement, and thus that its clinical application for the treatment of neurological disorders associated with abnormal PAC, such as PD, may be suitable.^14^ However, changes in PAC in the dyskinetic state remain enigmatic, and a better appreciation of PAC may yield a promising electrophysiological target for LID therapy.

Functional connectivity measures the similarity of specific physiological signals between different regions, reflecting the synchronization of field potentials and information transfer.^15^ It can also be regarded as an indicator of motor performance improvement, such as that achieved with L‐dopa treatment and DBS.^16^ Although fundamental experiments have revealed aberrant neural oscillations flow, a precise understanding of functional connectivity in the gamma band would contribute to the further exploration of the pathophysiological mechanism of LID and the prediction of the clinical efficacy of treatment.

While how chronic L‐dopa treatment results in LID is not fully understood, several possible mechanisms may underlie this effect. The widely accepted potential mechanism of the pathogenesis of LID is exceptional synaptic plasticity in the corticostriatal circuit due to the non‐physiological synthesis, release, and reuptake of DA from exogenous high‐dose L‐dopa metabolism.^17^, ^18^ However, much research has demonstrated that the pathogenesis of LID is related to not only dopaminergic receptors but also nondopaminergic receptors, such as serotonergic receptors,^19^ and various nondopaminergic medications are applied to manage such complications.^20^

Abnormal 5‐*hydroxytryptamine* (5‐HT) transmission is thought to be involved in various central nervous system (CNS) disorders, including anxiety, depression, schizophrenia, addiction, obsessive–compulsive disorder, PD, and Alzheimer's disease.^21^ In a non‐human primate study, Jiménez‐Sánchez et al.^22^ confirmed that the destruction of nigrostriatal dopaminergic neurons occurring in PD promotes the hyperinnervation of striatal 5‐HT fibers as a compensatory mechanism. 5‐HT neurons have been found to facilitate the conversion of exogenous L‐dopa to DA, the storage of DA in synaptic vesicles, and the release of DA in an activity‐dependent manner. As these neurons lack a negative feedback mechanism for the regulation of synaptic DA levels, these processes are highly uncontrolled, leading to persistent and prolonged abnormal DA release that significantly increases the striatal DA level and ultimately leads to LID.^23^ Several 5‐HT receptor agonists, such as NLX‐112,^24^ zonisamide,^25^ and buspirone,^26^ have been used to ameliorate dyskinesia in recent preclinical and clinical trials. Eltoprazine, a 5‐HT1A/B autoreceptor agonist, inhibits DA release from 5‐HT neurons and has been shown to effectively ameliorate dyskinesia in rats^27^ and patients^28^ with LID. Although eltoprazine has been shown to alleviate LID efficiently in a dose‐dependent manner, the changes in neural electrophysiology during its administration remain to be explored.

In this study, firstly, we investigated the accumulative effect of L‐dopa on neural oscillations in the corticostriatal projection. The LFPs from M1 and DLS were simultaneously recorded in Sham + saline, Sham + LB, PD + saline, and PD + LB groups for following assessment. Furthermore, we demonstrate the efficacy of eltoprazine in the alleviation of hyperkinesia in rats with LID and neural activity alterations to clarify its underlying therapeutic mechanism at the electrophysiological level for the first time. Lastly, we also explored the impact of eltoprazine on anti‐Parkinsonian efficacy of L‐dopa. Our research provided compelling evidence supporting future clinical application of eltoprazine in LID therapy.

## 2 MATERIALS AND METHODS

### 2.1 Animals

All experimental procedures were approved by the Institutional Animal Ethics Committee of Southern Medical University of China (LAEC‐2022‐146) and conducted under the National Institutes of Health Guidelines for the Care and Use of Laboratory Animals (NIH publications no. 8023, revised 1978). Forty‐five male Sprague–Dawley rats weighing 280–300 g were used for the experiment. The animals were purchased from the experimental animal center of Southern Medical University of China and housed under a standard 12/12‐h dark/light cycle (lights on at 08:00). To maintain the rats' body weight, their food intake was limited to 10 g/day.

### 2.2 Experimental procedure

#### 2.2.1 Experiment 1

Experiment 1 was performed with five rats. On day 1, 6‐hydroxydopamine (6‐OHDA; 4 μg/μL, 5 μL dissolved in 0.9% w/v saline; DR0610; HARVEYBIO) was injected into the right medial forebrain bundle (MFB) to induce a unilateral PD model. The open field test (OFT), cylinder test, and apomorphine (APO; 0.75 mg/kg, dissolved in 0.9% w/v saline, i.p.; Macklin) challenge were conducted on days 12, 13, and 14, respectively, to assess the success of PD modeling. Modeling was successful in five rats, which were then treated with L‐dopa (8 mg/kg, 2 mg/mL, dissolved in 0.9% w/v saline, i.p.; Macklin) and benserazide (12 mg/kg, 3 mg/mL, dissolved in 0.9% w/v saline, i.p.; Macklin; LB) for 14 consecutive days. On day 29–32, the rats were administered LB and eltoprazine (0, 0.4, 0.8, and 1.2 mg/kg, 1 mg/mL, i.p., respectively; Macklin) along with simultaneous video recording and abnormal involuntary movement scale (AIMs) scoring. The optimum eltoprazine concentration for follow‐up experiments was determined. After all experiments had been completed, the rats were sacrificed and brain tissue was harvested for histological analysis (Figure S1A).

#### 2.2.2 Experiment 2

Experiment 2 was performed with 52 rats. The rats were apportioned randomly to PD (*n* = 36) and sham (*n* = 16) groups. On day 1, 6‐hydroxydopamine (6‐OHDA; 4 μg/μL, 5 μL, dissolved in 0.9% w/v saline; HARVEYBIO) to induce unilateral PD lesions and saline vehicle (5 μL) to induce sham lesions were injected into the right medial forebrain bundle (MFB). The OFT, cylinder test, and APO (0.75 mg/kg, i.p.; Macklin) challenge were conducted on days 12, 13, and 14, respectively, to assess the success of PD modeling. PD was established successfully in 31 of the 36 rats. All 47 rats were implanted with microelectrodes on day 15 and recovery for 2 weeks postoperation. For 14 consecutive days, L‐dopa (8 mg/kg, 2 mg/mL, dissolved in 0.9% w/v saline, i.p.; Macklin) and benserazide (12 mg/kg, 3 mg/mL, dissolved in 0.9% w/v saline, i.p.; Macklin) were administered to 23 rats in the PD group (PD + LB group) and eight rats in the sham group (Sham + LB group); the remaining rats were treated with saline (2.4 mL, i.p.; Sham + saline and PD + saline groups, *n* = 8 each). Simultaneous video recording and abnormal involuntary movement scale (AIMs) scoring were conducted on days 29, 32, 35, and 38; LFP recording with synchronous video recording was performed on day 41. After the LB treatment period, rats in the PD + LB group were assigned randomly into LID + LB (*n* = 8), LID + LB + E (*n* = 9), and LID + E (*n* = 6) subgroups receiving LB, LB and eltoprazine (0.8 mg/kg, 1 mg/mL, dissolved in 0.9% w/v saline, i.p.; Macklin), or eltoprazine alone, respectively, for 7 consecutive days. Simultaneous video recording and AIMs scoring were performed on days 43, 46, and LFP recording with synchronous video recording was performed on day 49. Open field test was also performed on day 48 80 min after LB, LB plus eltoprazine, or eltoprazine injection. After all electrophysiological recordings had been completed, the rats were executed for histological analysis. One rat from the Sham + saline group was eliminated for missing the target.

### 2.3 Surgeries

For lesion creation, each rat's head was fixed in a stereotaxic apparatus after the induction of anesthesia with sodium pentobarbital (50 mg/kg, i.p.; Macklin). Unilateral lesions were created by injecting 6‐OHDA or saline into the right MFB according to the stereotaxic atlas of Paxinos and Watson (2007) at the following coordinates: anteroposterior (AP)—1.8 mm, mediolateral (ML)—2.0 mm, and dorsoventral (DV)—8.35 mm, below the skull surface.

For microelectrode implantation, two electrode arrays consisting of eight stainless‐steel Teflon‐insulated microwires (50‐μm diameter, 2 × 4 configuration with 150 μm spacing between microwires; Plexon) were implanted vertically in right primary motor cortex (M1) layers V and VI (AP +2.6 mm, ML −2.6 mm, DV −1.5 to 1.6 mm, below the dura) and the right dorsolateral striatum (DLS; AP 0 mm, ML −3.5 mm, DV –5.5 mm, below the dura), respectively. Two screws placed above the cerebellum and in contact with the dura were used as grounds, and ground wires were wrapped around them. The electrode arrays were secured to the cranium with screws and dental cement. Each rat received postoperative injections of penicillin (80,000 U/mL, 1 mL, i.p.) for 7 consecutive days after electrode implantation to prevent infection.

### 2.4 Behavioral tests

All rats were allowed to habituate to the behavior cages for 5 min before test initiation. For the OFT, each rat was placed in an opaque box (100 × 100 × 50 cm). It was permitted to move freely for 5 min, and the total distance and velocity were recorded for the assessment of locomotor activity with Noldus software (Noldus).

For the cylinder test, each rat was placed in an open transparent plastic cylinder, and 20 instances in which it used its forelimb to stand against the wall were recorded. Forelimb use was defined as the placement of the entire forepaw on the cylinder wall, indicating body support. The number of contacts with the forelimb contralateral to the lesioned side was calculated as a percentage of the total 20 contacts.

The APO challenge was performed to evaluate unilateral dopaminergic neuron depletion. The rats' rotations were counted 15 min after APO injection, and lesions were considered to be sufficient when rats made >30 contralateral rotations over a 5‐min period.

L‐dopa‐induced involuntary movement was scored using a rat dyskinesia scale.^27^ The severity of motor, forelimb, and orolingual dyskinesia was scored using the AIMs on a scale that motor, forelimb, and orolingual dyskinesias were each scored ranging from 0 to 4 (0, absence of abnormal movement; 1, dyskinesia occurring during <50% of the observational period; 2, dyskinesia occurring during ≥50% of the observational period; 3, constant dyskinesia that could be interrupted artificially; 4, constant dyskinesia that could not be interrupted). After L‐dopa plus benserazide (LB) injection, the rats were video‐recorded and observed for 180 min, and AIMs scores were recorded for 2‐min period during each of the 10‐ to 20‐min intervals (maximum AIMS score = 120). The interval between L‐dopa injection and the manifestation of abnormal involuntary movement was recorded as the response time (RT), as described previously.

### 2.5 Electrophysiological recording

Local field potentials in the M1 and DLS were recorded with a 128‐channel data acquisition system (Plexon). To record the baseline parkinsonian state, data were obtained 5 min before LB injection on day 29. LFPs were recorded in 11 5‐min sessions [1 before and 10 (at 20‐min intervals during a 180‐min period) after LB injection] on days 41 and 49. The data were collected at a sampling frequency of 1 kHz, amplified (300×), and bandpass filtered (0.5–1000 Hz). A ground wire was used for reference.

### 2.6 Histological analysis

Each rat was anesthetized with pentobarbital sodium (50 mg/kg, i.p.), and a 20‐μA current was passed through the recording electrodes for 30 s to mark the recording sites. Then, rat was then perfused transcardially with ice‐cold 0.9% w/v saline (400 mL) followed by 4% paraformaldehyde (400 mL). The brain was extracted and immersed in 4% paraformaldehyde for 24‐h post‐fixation, then dehydrated and paraffin embedded. Coronal sections (8 μm) through the M1, DLS, and SNc were cut on a paraffin microtome (Leica). To verify the recording sites, sections including electrode tracts were mounted on glass slides, stained with hematoxylin and eosin, and observed using a digital slide scanner (PANORAMIC; 3D Hitech Ltd).

### 2.7 Immunofluorescence staining

Immunofluorescence staining of tyrosine hydroxylase (TH) in the SNc was performed to measure DA depletion. All sections were processed using the same protocol and chemical reagents. The sections were placed in a dry oven at 65°C for 60 min, then washed to remove the paraffin and rehydrate the tissue in dimethylbenzene (10 min, three times); 100%, 95%, 85%, and 75% ethanol (5 min each); and double‐distilled water (5 min, three times). Then, the sections were immersed in citrate antigen retrieval solution (Beyotime) and microwaved until boiling. Maintain a consecutive boiling for 15 min, then cooled to room temperature and washed three times (5 min each) with phosphate‐buffered saline (PBS). The sections were incubated for 1 h at room temperature with 5% normal donkey serum (017‐000‐121‐E; Jackson) in PBS containing 0.03% Triton X‐100, and left overnight at 4°C in PBS containing rabbit anti‐TH (extracellular) polyclonal antibody (1:500, EP1532Y; Abcam). After rinsing three times with PBS, the sections were incubated at room temperature for 2 h in PBS containing Alexa Fluro 594 donkey anti‐rabbit IgG secondary antibody (1:500, 711‐585‐152; Jackson). After several rinses, the sections were incubated at room temperature for 3 min in DAPI (C0065‐10; Solarbio).

The labeling intensity in each area was quantified as the index of light attenuation with respect to the background (neighboring corpus callosum) using the standard ImageJ (National Institutes of Health) program. The amount of TH+ cells was calculated according to the optical fractionator principle using ImageJ.

### 2.8 Data analysis

#### 2.8.1 Data preprocessing

The Fieldtrip Toolbox^29^ was used to preprocess the data. Using ft_definetrial, data from the 5‐min LFP sessions were divided into 300 1‐s segments. Then, artifacts were identified and removed using ft_artifact_zvalue and ft_rejectartifact.

#### 2.8.2 Spectral analysis of LFPs

Power spectrum density (PSD) was estimated based on fast Fourier transform using the ft_freqanalysis function of Fieldtrip. In total, 120 1‐s processed data segments were used to calculate PSDs with cfg.foilim = 1:1:150 (target frequency range, 1–150 Hz), tapsmofrq (amount of spectral smoothing achieved through multi‐tapering) = 2, and a Hanning window.

#### 2.8.3 Aperiodic‐adjusted power of beta and gamma oscillations

We analyzed the aperiodic‐adjusted beta power in the baseline (day‐29) parkinsonian state and the gamma oscillations power at 80 min after LB administration on day 41 using the Fitting Oscillations & One Over F (FOOOF) toolbox.^30^ The Matlab package was utilized to separate periodic and aperiodic components from the spectral results, according to our previous research.^31^ The PSD for each frequency $f\;\left[\;P\left(f\right)\right]$ was expressed as: $P\left(f\right)=L\left(f\right)+\sum_{n}^{\;}G_{n}\left(f\right),$ where $L\left(f\right)$ is the aperiodic component and $G_{n}\left(f\right)$ is the Gaussian distribution. The periodic component was parameterized as a mixture of Gaussian distributions: $G_{n}\left(f\right)=\alpha *\exp\left(\frac{-{\left(f-c\right)}^{2}}{2*\omega^{2}}\right)$, where α is the height of the peak (power) above the aperiodic component, c is the center frequency of the peak (peak frequency), ω is the width of the peak (bandwidth), and f is the array of frequency values. The aperiodic component was also parameterized using a Lorentzian function as $L\left(f\right)=b-\log\left(k+f^{\chi }\right)$, where b is the broadband offset, k is the “knee,” and χ is the exponent of the aperiodic fit. The parameters of aperiodic‐adjusted beta and gamma power were set as: fitted frequency range = [1, 50] and [60, 140], respectively; peak width limits = [0, 12]; and fixed (fitted with no knee parameter) aperiodic mode, considering the narrow frequency range. All models used in further analyses met the condition of $R^{2}$ > 0.95.

#### 2.8.4 PAC

The Eeglab toolbox (2021.0, a free Matlab toolbox) was used to calculate and visualize theta–gamma PAC. First, a comodulogram of PAC was drawn using the modulation index (MI) measure for multiple pairs of phase–frequency and amplitude–frequency bands. Next, PAC was quantified using the mean vector length MI method among three phase frequencies at 1–10 Hz and 20 amplitude frequencies at 60–120 Hz. Whether enhanced broadband gamma oscillation in the DLS was modulated by the theta oscillation phase was examined.

#### 2.8.5 Functional connectivity

Coherence was identified by measuring the similarity of LFPs across different frequencies, with values ranging from 0 (no linear association) to 1 (perfect similarity). It was used to quantify the functional connectivity between the M1 and DLS with the ft_connectivity analysis function of Fieldtrip. The calculation parameters were cfg.foilim = 1:1:150 and cfg.tapsmofrq = 5.

The phase‐locking values (PLVs) were used to quantify phase‐dependent functional connectivity between the M1 and DLS. It was calculated using the filtfilt function (Matlab 9.3.0.713579, R2017b, MathWorks) and preprocessed M1 and DLS data from frequency bands at 20–35 and 80–110 Hz. The instantaneous phases were extracted from the filtered data by Hilbert transformation. Differences between these phases were normalized as PLVs. Low PLVs between distinct regions were taken to represent unsynchronized signals, and high values were taken to indicate great extents of synchronization.

### 2.9 Statistical analysis

All data are reported as means with standard errors of the mean. All data were tested for normal distribution before statistical analysis. The data exhibited a normal distribution was further undergone parametric test while the data did not exhibit a normal distribution was further analyzed via a nonparametric equivalent Kolmogorov–Smirnov test. Data from the Sham + saline, Sham + LB, PD + saline, and PD + LB groups were subjected to two‐way analysis of variance (ANOVA), with Tukey's multiple comparison test applied to significant results. Student's *t* test and Kolmogorov–Smirnov test were used to compare behavioral test and *immunofluorescence* results between rats in the Sham and PD groups; AIMs scores, RTs between the LID + LB and LID + LB + E groups. Repeated measures (RM) one‐way ANOVA with Tukey's multiple comparison test was applied to compare the effect of eltoprazine intervention in different concentration. One‐way ANOVA with Tukey's multiple comparison test was conducted to analyze the total motor distance and velocity in OFT on day 48; PSDs, PAC, coherence, and PLVs among LID + LB, LID + LB + E, and LID + E groups. Correlations between beta and gamma power were assessed using Pearson correlation analysis. For PSDs and coherence, areas under the curve for beta‐ and gamma‐band oscillations were calculated. *p* values < 0.05 were considered to be significant. The analyses were conducted with GraphPad Prism 9.0 software (GraphPad Software).

## 3 RESULTS

### 3.1 PD model establishment

**FIGURE 1:**
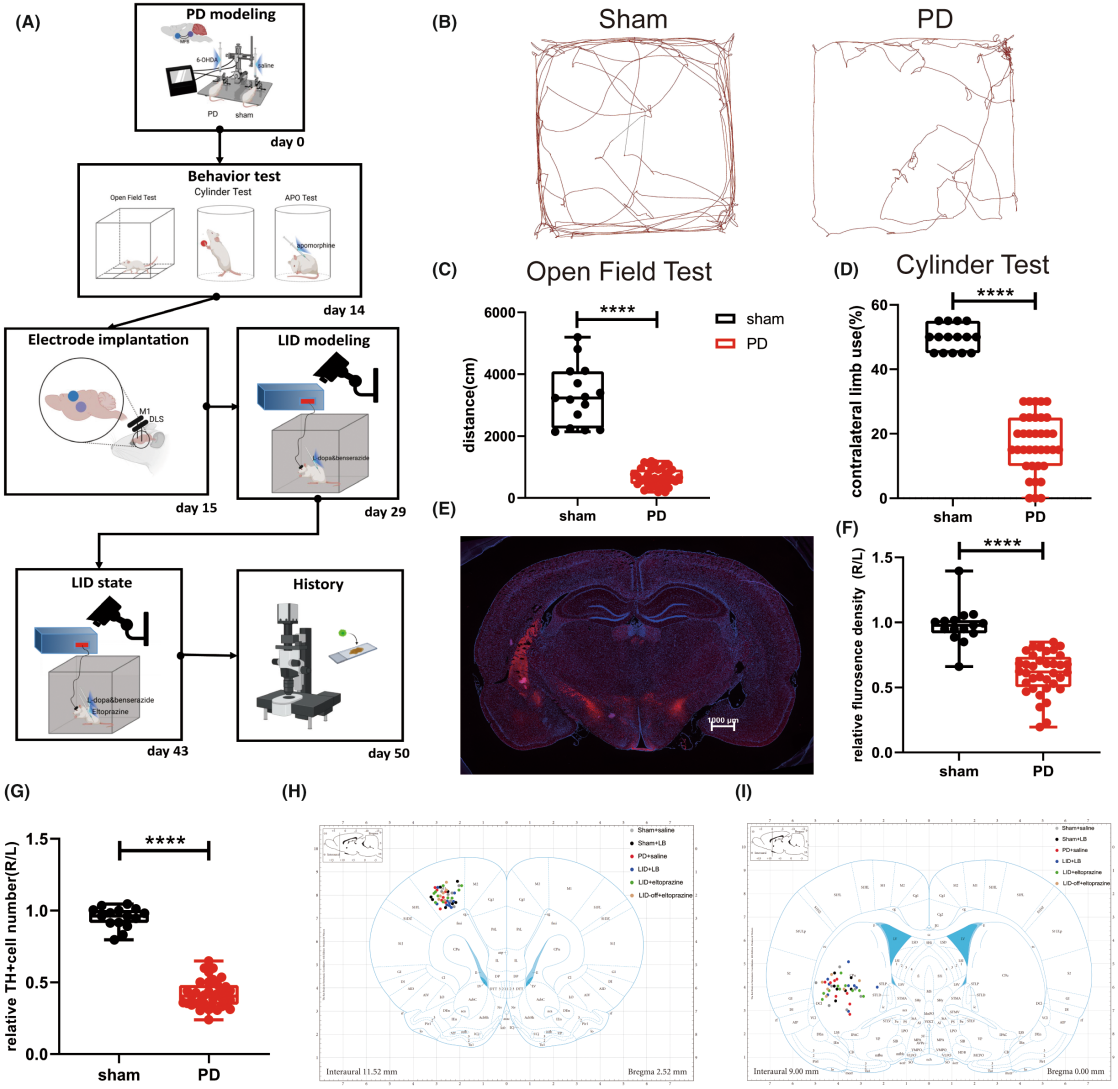
Evaluation of PD modeling. (A) Schematic diagram of the Experimental 2 procedure. (B, C) Representative open field test motion trails and total distance traveled. (D) Contralateral limb use in the cylinder test. (E) Representative TH *immunofluorescence* labeling of DA neurons in the SNc in rats with PD. (F, G) Relative TH *immunofluorescence* density and number of TH+ cells in the SNc. (H, I) Electrode implantation sites in the M1 and DLS. Data are means ± SEMs. ****p < 0001, Student's *t* and Kolmogorov–Smirnov test.

PD was established successfully in 25 of the 29 rats (Figure 1A). Rats with 6‐OHDA lesions showed reduced total distance and velocity on the OFT (Student's *t* test, total distance, *p* < 0.0001; velocity, *p* < 0.0001; Figure 1B,C, Figure S1B) and impaired contralateral limb use in the cylinder test (Student's *t* test, *p* < 0.0001; Figure 1D), consistent with parkinsonian bradykinesia and akinesia symptoms. Rats with PD showed prominent degeneration of TH‐positive cells which marking dopaminergic neurons depletion in the SNc, with decreased fluorescence intensity (Kolmogorov–Smirnov test, *p* < 0.0001) and cell numbers (Student's *t* test, *p* < 0.0001; Figure 1E–G).

### 3.2 L‐dopa‐induced exaggerated gamma oscillation in the M1 and DLS

**FIGURE 2:**
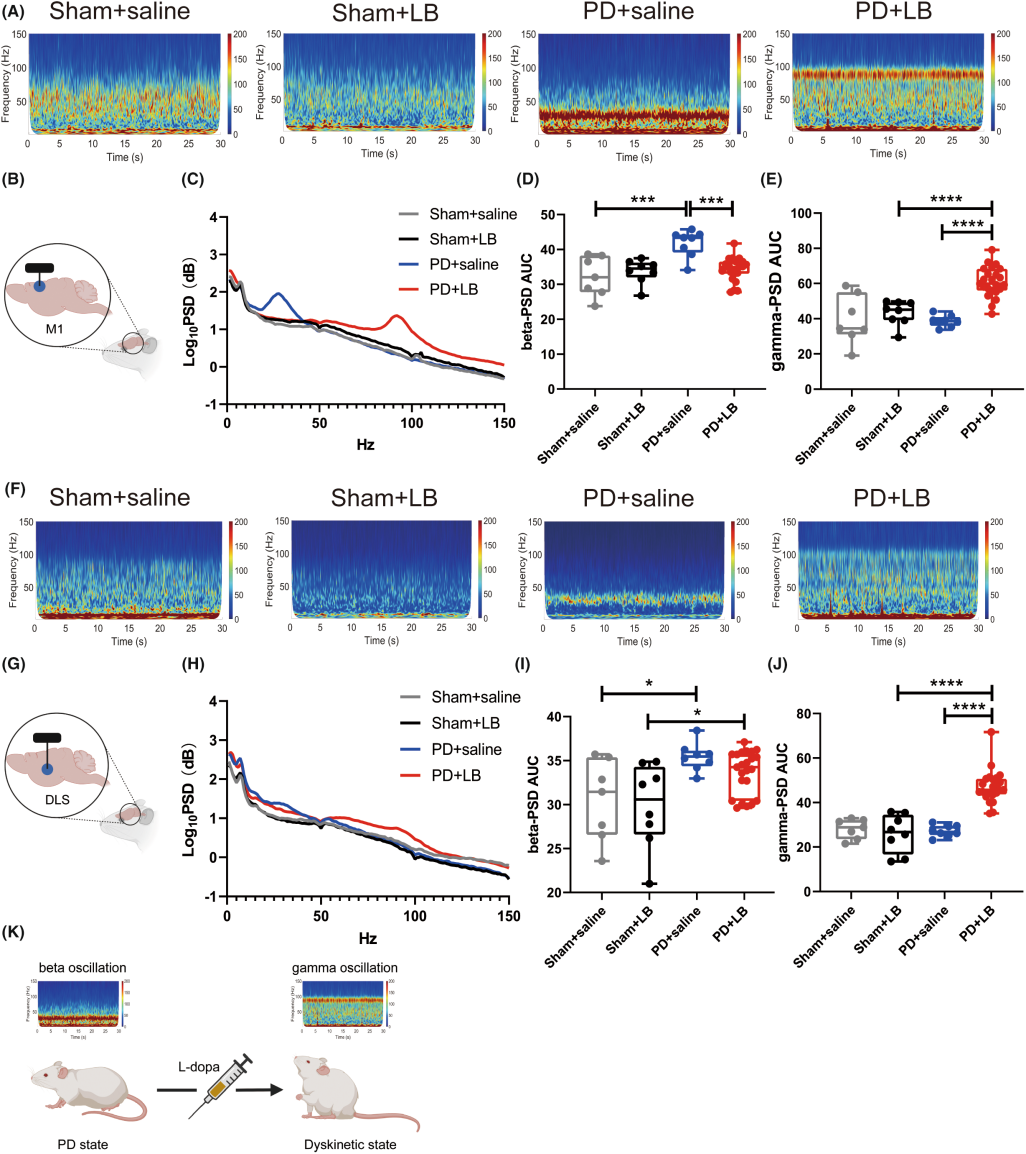
Exaggerated gamma oscillation elicited by LB in the M1 and DLS. (A, F) Representative M1 and DLS PSD spectrum plots. (B, G) M1 and DLS locations. (C, H) Power spectral density of LFPs in the M1 and DLS. (D, E, I, J) Summaries of beta‐ and gamma‐band oscillation in the M1 and DLS. *p < 0.05, ***p < 0.001, ****p < 0001, two‐way ANOVA followed by Tukey's multiple comparison test.

PSDs were estimated using LFPs at 80 min, corresponding to peak dyskinesia after LB administration, on day 41. In the M1, the beta oscillations power was greater in the PD + saline group than in the Sham + saline and PD + LB groups (two‐way ANOVA: LB: *F*_1,42_ = 5.633, *p* = 0.0223; PD: *F*_1,42_ = 15.28, *p* = 0.0003; LB × PD interaction: *F*_1,42_ = 12.66, *p* = 0.0009; Tukey's test, both *p* = 0.0001; Figure 2A–D). Narrowband gamma oscillations were exaggerated in the PD + LB group relative to the Sham + LB and PD + saline groups (two‐way ANOVA: LB: *F*_1,42_ = 20.63, *p* < 0.0001; PD: *F*_1,42_ = 8.520, *p* = 0.0056; LB × PD interaction, *F*_1,42_ = 10.30, *p* = 0.0025; Tukey's test: both *p* < 0.0001; Figure 2E). In the DLS, the PD + saline group showed enhanced beta activities (two‐way ANOVA: LB: *F*_1,42_ = 1.228, *p* = 0.2742; PD: *F*_1,42_ = 16.87, *p* = 0.0002; LB × PD interaction, *F*_1,42_ = 0.2845, *p* = 0.5966; Tukey's test, Sham + saline, *p* = 0.0249; Figure 2F–I) while PD + LB group exhibited augment gamma oscillations (at broader bands than in the M1; two‐way ANOVA: LB: *F*_1,42_ = 15.80, *p* = 0.0003; PD: *F*_1,42_ = 20.66, *p* < 0.0001; LB × PD interaction: *F*_1,42_ = 24.67, *p* < 0.0001; Tukey's test, both *p* < 0.0001 vs. Sham + LB and PD + saline; Figure 2J) among groups.

### 3.3 Effects on gamma oscillation power

**FIGURE 3:**
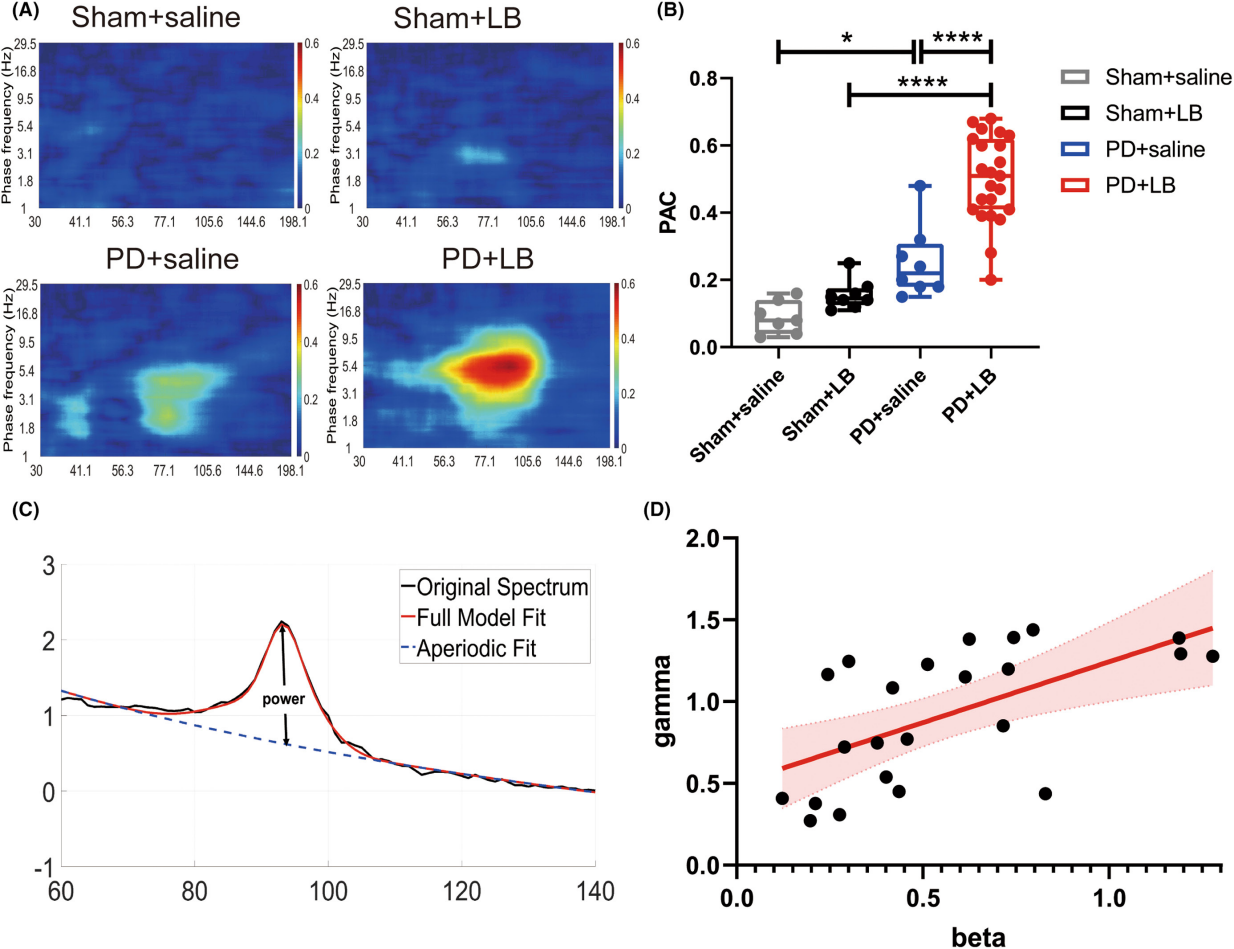
Effects on gamma oscillation power. (A) Representative comodulograms of theta–gamma PAC in DLS. (B) Summary of theta–gamma PAC in the DLS. (C) Schematic diagram showing beta and gamma power extraction with the FOOOF tool. (D) Correlation between beta and gamma power. Data are means ± SEMs. *p < 0.05, ****p < 0.0001, two‐way ANOVA followed by Tukey's multiple comparison test.

Theta–gamma PAC in the DLS was greater in the PD + saline group than in the Sham + saline group (Tukey's test, *p* = 0.0226), and greater in the PD + LB group than in the Sham + LB and PD + saline groups (two‐way ANOVA: LB: *F*_1,42_ = 20.36, *p* < 0.0001; PD: *F*_1,42_ = 52.88, *p* < 0.0001; LB × PD interaction, *F*_1,42_ = 6.614, *p* = 0.0137; Tukey's test, both *p* < 0.0001; Figure 3A,B).

It has been proved that beta‐band power was related to the severity of PD symptoms, but the correlation between beta and gamma power remains obscure. We analyzed the aperiodic‐adjusted power of beta rhythm of parkinsonian state baseline on day 29 and power of gamma oscillations at 80 min after LB administration on day 41 by Fitting Oscillations & One Over f (FOOOF) toolbox (Figure 3C). The beta and gamma power correlated positively (*r* = 0.6026, *p* = 0.0023; Figure 3D).

### 3.4 Functional connectivity in the cortico–basal ganglia circuit in the PD and LID states

**FIGURE 4:**
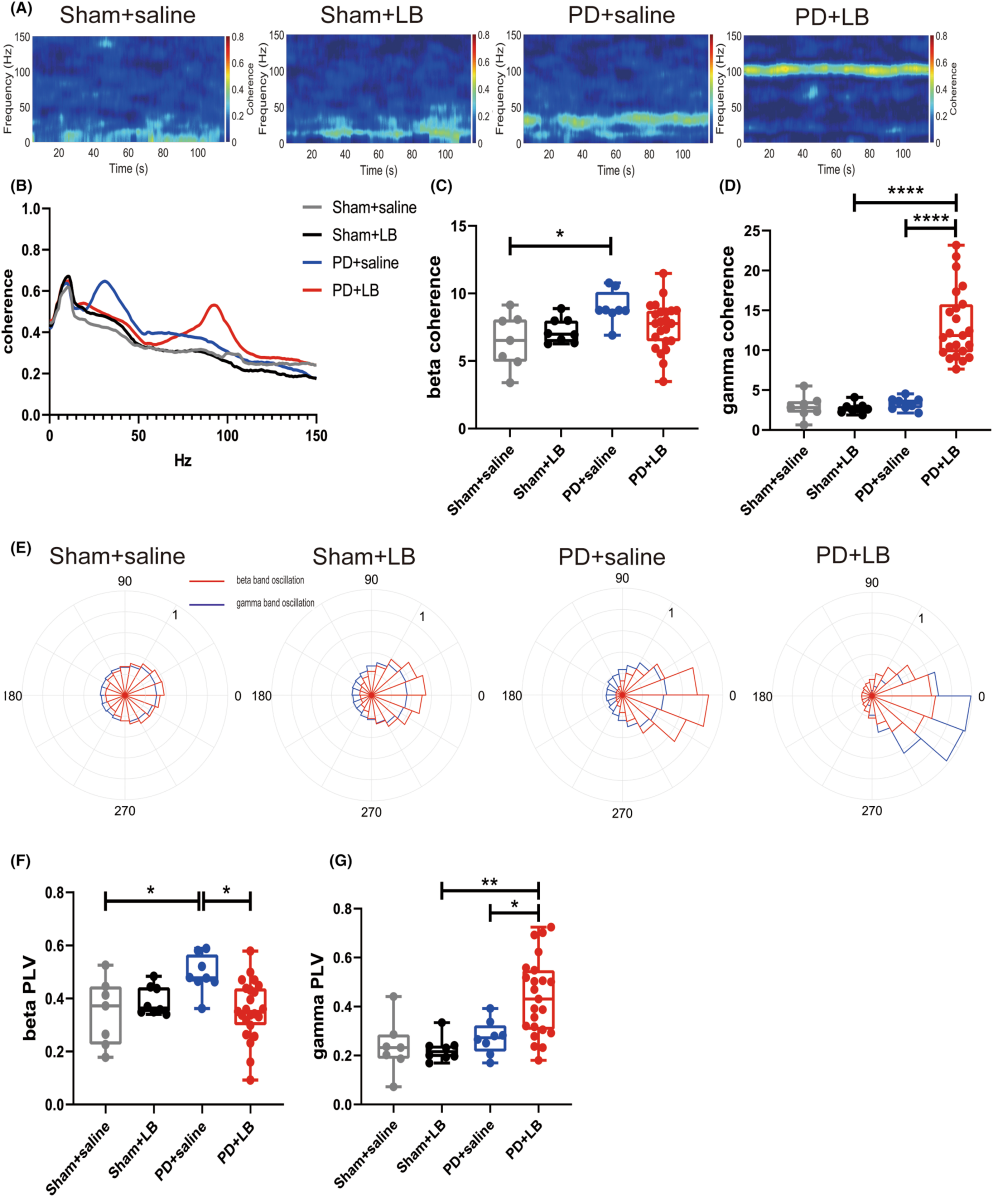
Functional connectivity in the cortico–basal ganglia circuit in the PD and LID states. (A) Representative spectrum plots of coherence between the M1 and DLS. (B) M1–DLS coherence. (C, D) Summaries of coherence in the beta and gamma bands. (E) Representative polar histograms of phase locking between the M1 and DLS; red lines represent beta oscillation and blue lines represent gamma oscillation; (F, G) Summaries of beta‐ and gamma‐band PLVs. Data are means ± SEMs. *p < 0.05, **p < 0.01, two‐way ANOVA followed by Tukey's multiple comparison test.

To further investigated the effect of LB administration on cortical–striatal circuit in different states, coherence which reveals a connectivity based on amplitude was first examined between M1 and DLS (Figure 4A). Beta‐band coherence was enhanced in the PD + saline group compared with the Sham + saline group (two‐way ANOVA: LB: *F*_1,42_ = 0.3448, *p* = 0.5602; PD: *F*_1,42_ = 6.916, *p* = 0.0119; LB × PD interaction, *F*_1,42_ = 4.088, *p* = 0.0496; Tukey's test, *p* = 0.0245; Figure 4B,C). Stronger gamma‐band coherence was detected in the PD + LB group than in other groups (two‐way ANOVA: LB: *F*_1,42_ = 20.08, *p* < 0.0001; PD: *F*_1,42_ = 24.96, *p* < 0.0001; LB × PD interaction, *F*_1,42_ = 21.42, *p* < 0.0001; Tukey's test: both *p* < 0.0001; Figure 4D). Similarly, beta‐band PLVs were higher in the PD + saline group than in the PD + LB and Sham + saline groups (two‐way ANOVA: LB: *F*_1,42_ = 1.868, *p* = 0.1790; PD: *F*_1,42_ = 2.769, *p* = 0.1036; LB × PD interaction, *F*_1,42_ = 7.368, *p* = 0.0096; Tukey's test: PD + LB, *p* = 0.0107; Sham + saline, *p* = 0.0377; Figure 4E). Gamma‐band PLVs were higher in the PD + LB group than in the Sham + LB group (two‐way ANOVA: LB: *F*_1,42_ = 3.382, *p* = 0.0730; PD: *F*_1,42_ = 8.764, *p* = 0.0050; LB × PD interaction, *F*_1,42_ = 4.421, *p* = 0.0415; Tukey's test, *p* = 0.0011; Figure 4F,G).

### 3.5 Effect of eltoprazine on the motor performance and neural activities of rats with LID

**FIGURE 5:**
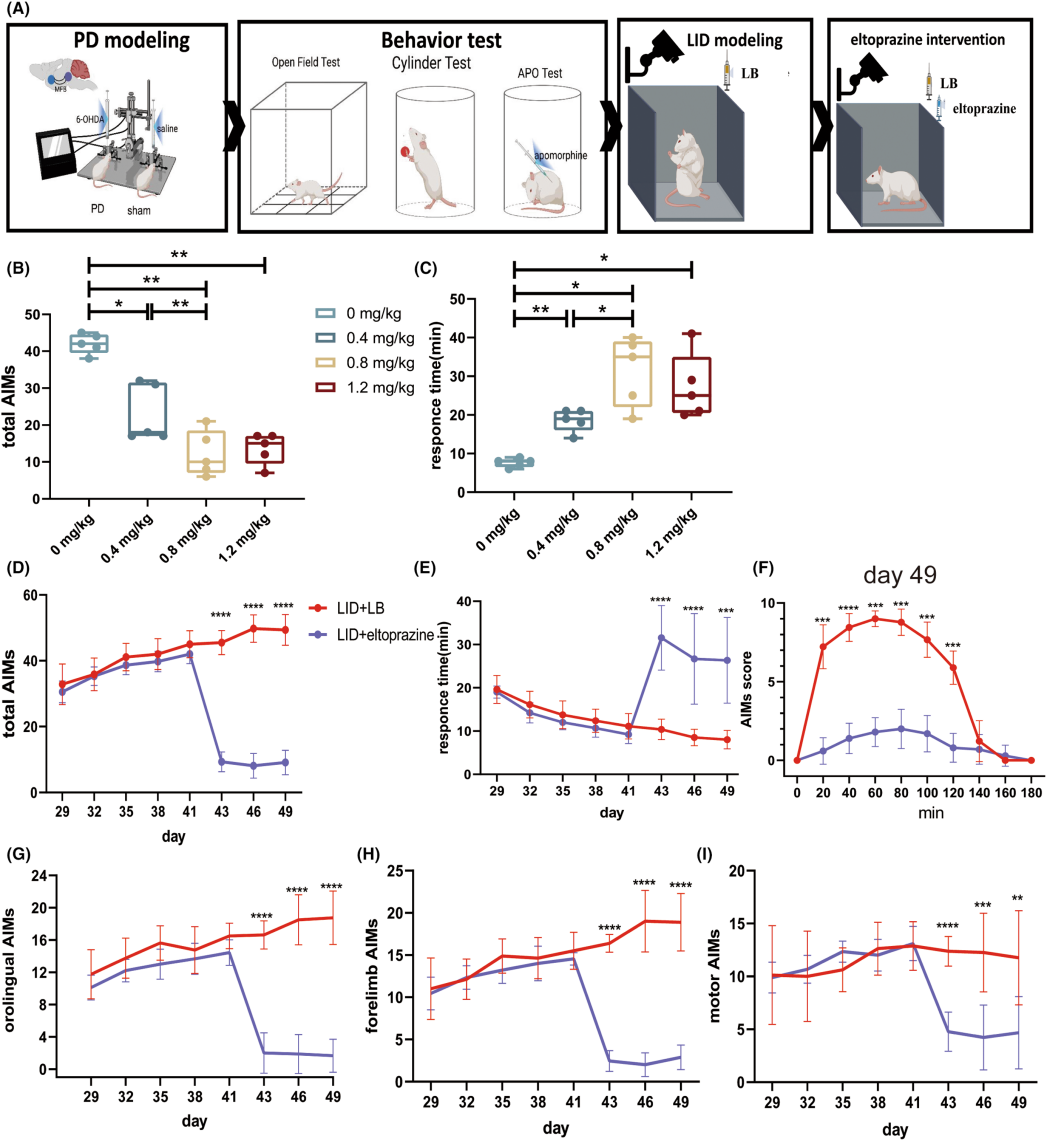
Eltoprazine ameliorated motor performance in rats with LID. (A) Schematic diagram of the Experimental 1 procedure. (B, C) Total AIMs and response times scores after LB administration and eltoprazine intervention in different dosage. (D, E) Total AIMs scores and response times after LB priming, overall. (F) Total AIMs scores after LB priming on day 49. (G–I) Orolingual, forelimb, and motor AIMs scores after LB priming. Data are means ± SEMs. *p < 0.05, **p < 0.01, ***p < 0.001, ****p < 0.0001, Student's *t* and Kolmogorov–Smirnov test.

After 14 days of consecutive LB administration, the LID model was established stably. The eltoprazine intervention ameliorated dyskinetic symptoms, declining AIMs scores (RM one‐way ANOVA, *F* = 35.49, *p* = 0.0009; Tukey's test: 0 vs. 0.4, 0.8, and 1.2 mg/kg, *p* = 0.0139, *p* = 0.0016, *p* = 0.0010, respectively; 0.4 vs. 0.8 mg/kg, *p* = 0.0047; Figure 5B) and delaying RTs (RM one‐way ANOVA, *F* = 11.79, *p* = 0.0151; Tukey's test: 0 vs. 0.4, 0.8, and 1.2 mg/kg, *p* = 0.0083, *p* = 0.0174, *p* = 0.0211, respectively; 0.4 vs. 0.8 mg/kg, *p* = 0.0356; Figure 5C). AIMs scores were much lower and RTs were much longer with 1.2 and 0.8 mg/kg eltoprazine than with 0.4 mg/kg, with no difference between 0.8 and 1.2 mg/kg. Thus, 0.8 mg/kg eltoprazine was used in follow‐up behavioral and electrophysiological analyses.

Subsequently, above‐mentioned PD + LB group was randomly separated into LID + LB, LID + LB + E, and LID + E subgroups receiving LB, LB and eltoprazine, or eltoprazine alone, respectively, to investigate the effect of eltoprazine on neural activities' alterations induced by LB and exclude the own effect of eltoprazine.

No dyskinetic symptoms appeared in the LID + E group in the last 7 days (Figure S1B), while the LID + LB + E group had a greatly reduced AIMs score (Student's *t* test, *p* < 0.0001 on days 43, 46, and 49; Figure 5D) and delayed RTs (Student's *t* test, *p* < 0.0001 on day 43, Kolmogorov–Smirnov test, *p* < 0.0001 on day 46, Student's *t* test, *p* = 0.0001 on day 49; Figure 5E) compared to LID + LB group. In addition, the AIMs score declined significantly at each 20‐min interval from 20 to 120 min following a single eltoprazine and LB injection on days 43 and 49 (day 49, 20 min, Kolmogorov–Smirnov test, *p* = 0.0002, 40 min, Student's *t* test, *p* < 0.0001, 60–120 min, Kolmogorov–Smirnov test, *p* = 0.0002; day 43, 20–40 min, Kolmogorov–Smirnov test, *p* = 0.0002, 60–80 min, Student's *t* test, *p* < 0.0001, 100–120 min, Kolmogorov–Smirnov test, *p* = 0.0002; Figure 5F, Figure S1C). AIMs subscale scores showed the same declining trend on days 43, 46, and 49 (orolingual, Student's *t* test, *p* < 0.0001 on day 43, Kolmogorov–Smirnov test, *p* < 0.0001 on day 46 and 49; forelimb, Kolmogorov–Smirnov test, *p* < 0.0001 on day 43 and 46, Student's *t* test, *p* < 0.0001 on day 49; motor, *p* < 0.0001 on day 43, *p* = 0.0002 on day 46, *p* = 0.002 on day 49; Figure 5G–I).

Locomotor activity of LID + LB, LID + LB + E, and LID + E groups rats was evaluated by OFT 80 min after LB/LB plus eltoprazine/eltoprazine administration on day 48. LID + LB + E group rats exhibited improved locomotion with increased movement distance and velocity relative to the others on day 48 (one‐way ANOVA, total distance: *F*_2,20_ = 5.810, *p* = 0.0102; Tukey's test: LID + LB vs. LID + LB + E, *p* = 0.0076; LID + LB + E vs. LID + E, *p* = 0.0046; velocity: *F*_2,20_ = 8.287, *p* = 0.0024; Tukey's test: LID + LB vs. LID + LB + E, *p* = 0.0104; LID + LB + E vs. LID + E, *p* = 0.0047; Figure 1F–H).

**FIGURE 6:**
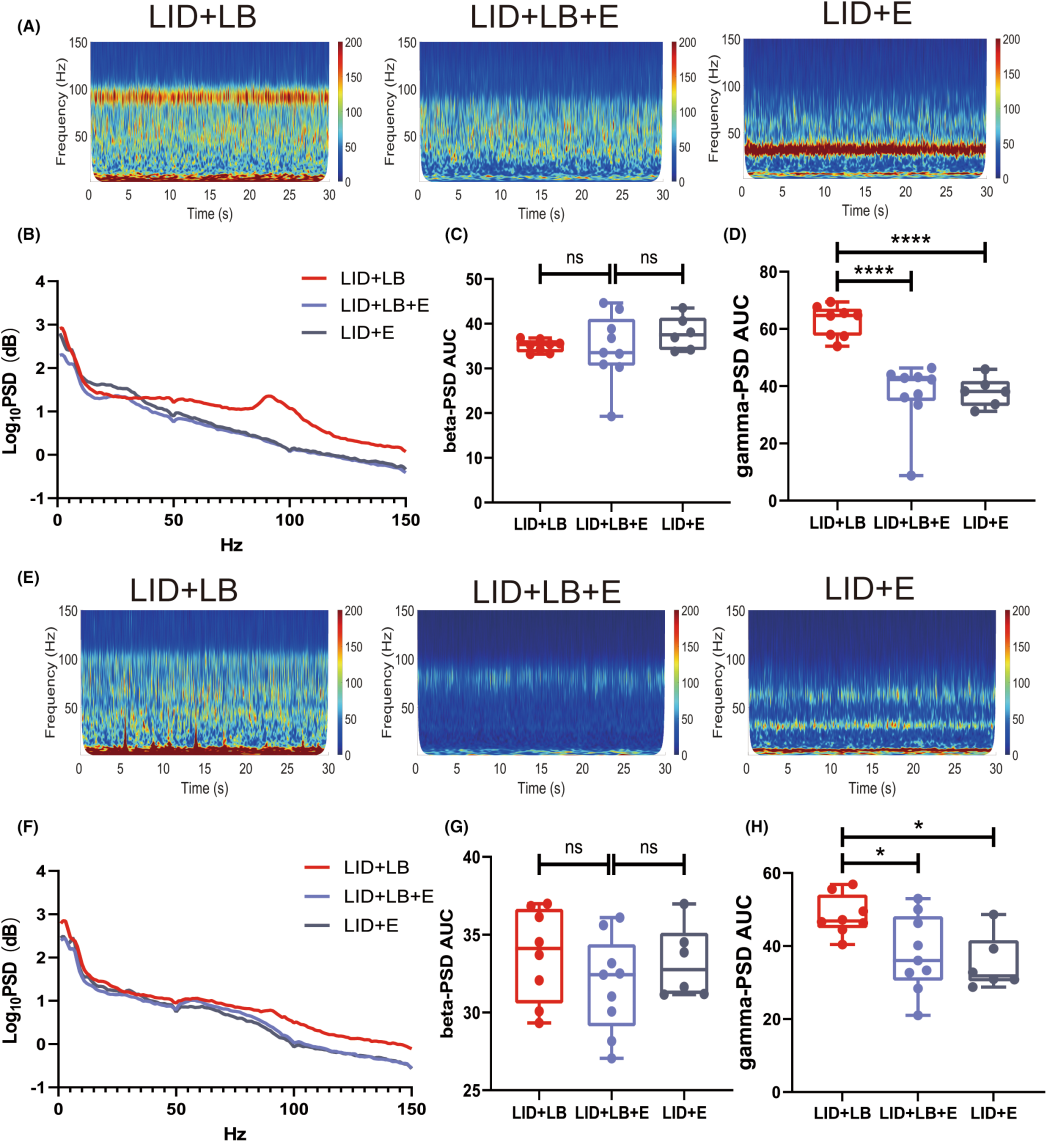
Effect of eltoprazine on neural activities of rats with LID. (A, E) Representative M1 and DLS PSD spectrum plots. (B, F) Power spectral densities of LFPs in the M1 and DLS. (C, D, G, H) Summaries of beta‐ and gamma‐band oscillation extracted from the M1 and DLS. Data are means ± SEMs. *p < 0.05, **p < 0.01, ***p < 0.001, ****p < 0.0001, one‐way ANOVA followed by Tukey's multiple comparison test.

**FIGURE 7:**
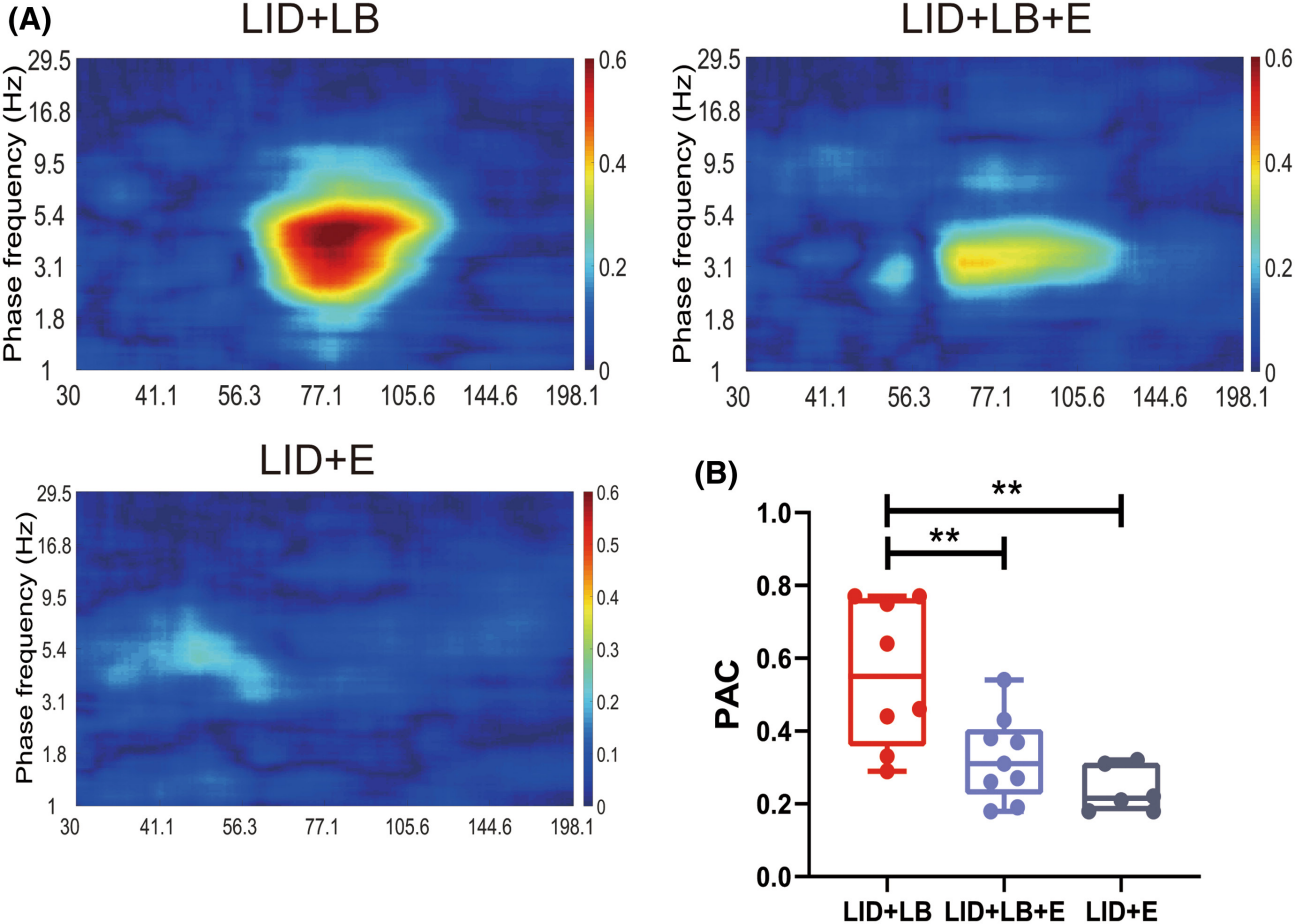
Effect of eltoprazine on theta–gamma PAC of rats with LID. (A) Representative images of theta–gamma PAC. (B) Comparison of theta–gamma PAC between LID + LB, LID + LB + E, LID + E groups rats. Data are means ± SEMs. *p < 0.05, **p < 0.01, ***p < 0.001, ****p < 0.0001, one‐way ANOVA followed by Tukey's multiple comparison test.

**FIGURE 8:**
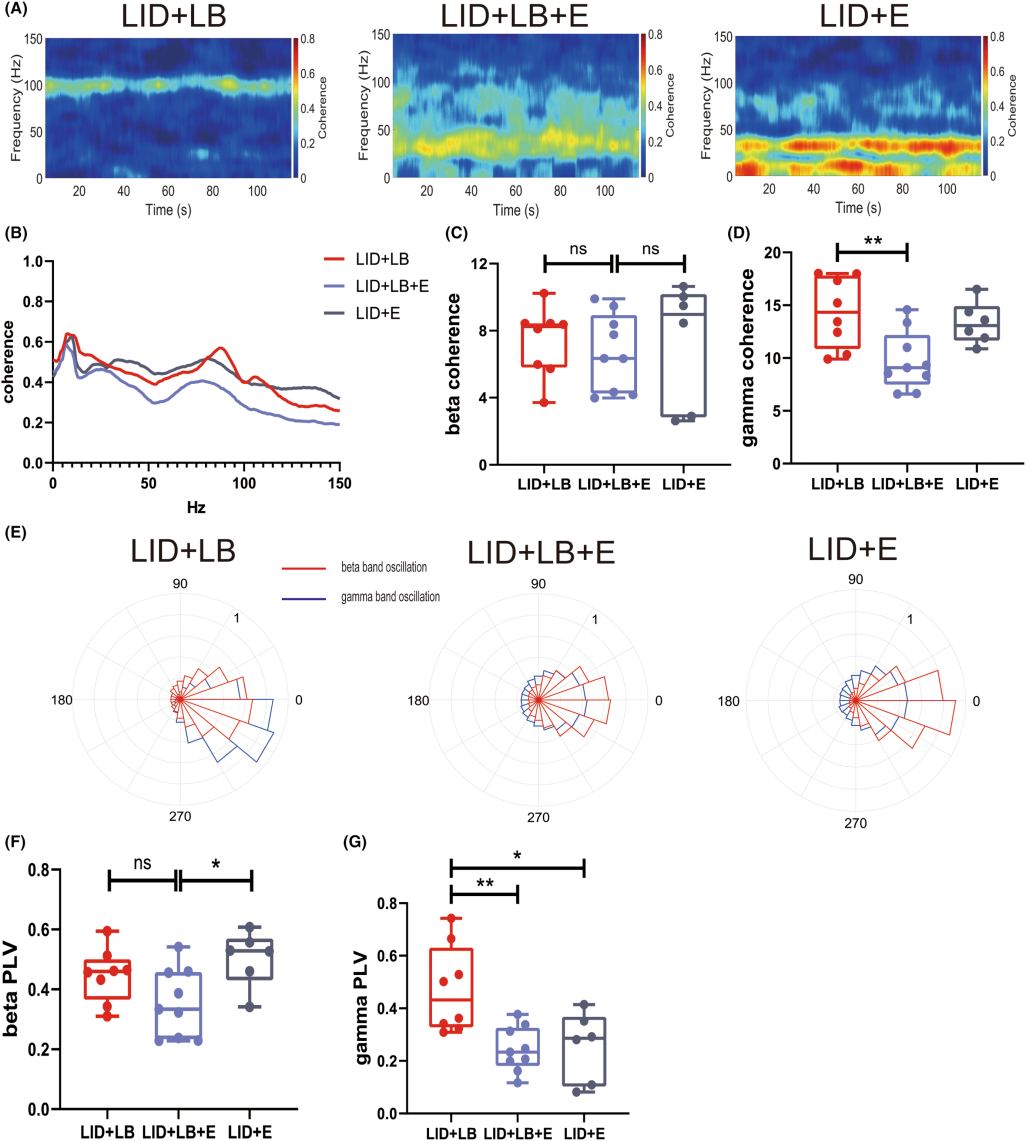
Neural electrophysiological alterations in the cortico–basal ganglia circuit after eltoprazine administration. (A) Representative spectrum plots of coherence between the M1 and DLS. (B) M1–DLS coherence. (C, D) Summaries of beta‐ and gamma‐band coherence. (E) Representative plots of phase locking between the M1 and DLS. (F, G) Summaries of beta‐ and gamma‐band PLVs. Data are means ± SEMs. *p < 0.05, **p < 0.01, ***p < 0.001, ****p < 0.0001, one‐way ANOVA followed by Tukey's multiple comparison test.

Electrophysiological alterations were analyzed after eltoprazine intervention on day 49. Compared with the LID + LB group, both LID + LB + E and LID + E groups exhibited a reduced gamma oscillation power (one‐way ANOVA, M1: *F*_2,20_ = 23.83, *p* < 0.0001; Tukey's test: both *p* < 0.0001; DLS: *F*_2,20_ = 5.284, *p* = 0.0144; Tukey's test: LID + LB + E, *p* = 0.0425; LID + E, *p* = 0.0208; Figure 6A,B,D,E,F,H) with no difference in power of beta oscillations (one‐way ANOVA, M1: *F*_2,20_ = 0.7807, *p* = 0.4715; Tukey's test: LID + LB + E, *p* = 0.9788; LID + E, *p* = 0.5909; DLS: *F*_2,20_ = 1.023, *p* = 0.3777; Tukey's test: LID + LB + E, *p* = 0.3722; LID + E, *p* = 0.9483; Figure 6C,G) on day 49 in the M1 and DLS. In addition, no difference in both gamma‐ and beta‐band oscillations power was observed between LID + LB + E and LID + E groups in either the M1 or DLS (Tukey's test: M1, gamma‐band, *p* = 0.9823; beta‐band, *p* = 0.4683; DLS, gamma‐band, *p* = 0.8142; beta‐band, *p* = 0.6161). The eltoprazine intervention suppressed the upregulated theta–gamma PAC in the DLS (one‐way ANOVA, *F*_2,20_ = 9.725, *p* = 0.0011; Tukey's test: LID + LB + E, *p* = 0.0092; LID + E, *p* = 0.0014; Figure 7A,B). The enhanced gamma‐band coherence and PLVs between M1 and DLS in the dyskinetic state were declined in LID + LB + E and LID + E groups (one‐way ANOVA, coherence, *F*_2,20_ = 6.252, *p* = 0.0078; Tukey's test: LID + LB + E, *p* = 0.0081; PLVs, *F*_2,20_ = 7.724, *p* = 0.0033; Tukey's test: LID + LB + E, *p* = 0.0048; LID + E, *p* = 0.0153; Figure 8A,B,D,E,G). The beta‐band coherence did not differ among groups (one‐way ANOVA, *F*_2,20_ = 0.1571, *p* = 0.8557; Tukey's test: LID + LB + E vs. LID + LB, *p* = 0.8725; LID + E vs. LID + LB, *p* = 0.9999; Figure 8C). The beta‐band PLVs were unchanged between LID + LB and LID + LB + E groups (one‐way ANOVA, *F*_2,20_ = 4.182, *p* = 0.0304; Tukey's test: *p* = 0.1721; Figure 8F), but it was strengthened in LID + E group (Tukey's test: *p* = 0.0287). However, no alterations in theta–gamma PAC in the DLS, coherence in both gamma‐ and beta‐band as well as PLVs in gamma band were detected between LID + LB + E and LID + E groups (Tukey's test: theta–gamma PAC, *p* = 0.4791; coherence: gamma‐band, *p* = 0.0628; beta‐band, *p* = 0.8967; PLVs, *p* = 0.9857).

## 4 DISCUSSION

To better understand the pathological mechanism underlying LID at the electrophysiological level, we investigated the effect of L‐dopa on gamma oscillations in the cortico–basal ganglia circuit and the factors influencing gamma oscillations power. We also examined the electrophysiological alterations occurring after eltoprazine intervention in rats with LID. Gamma activities were spatially dependent, with different characteristics and influencing factors in distinct regions; gamma oscillations were narrowband in the M1 and broadband in the DLS. The exaggerated aperiodic‐adjusted gamma activities power correlated positively with the aperiodic‐corrected power of beta rhythm in M1, while striatal gamma oscillations were modulated by enhanced theta–gamma PAC in the DLS. LB administration increased gamma‐band coherence and PLVs. Eltoprazine regulated the aberrant neural activities elicited by LB injection, ameliorating dyskinetic symptoms.

Most neural electrophysiological studies of LID have focused on gamma oscillations due to its potential biomarker function. Narrowband and broadband patterns of gamma oscillations are distinct, with different characteristics, functions, and nuclei; the former can be elicited by dopaminergic therapy or DBS of the M1, while the latter is associated with motor processing in the subcortical nucleus.^32^ In keeping with the previous researches, we also found exaggerated gamma oscillations arose in different appearance in distinct regions that the gamma oscillations in M1 showed a narrowband manner while that in DLS had a broadband appearance. Gamma oscillations are considered to have a prokinetic function, in patients with PD, enhanced broadband gamma oscillations correlate positively with motor improvement during voluntary movement.^13^ However, excessively exaggerated narrowband gamma oscillations in cortico–basal ganglia–thalamic neural circuits may be related to involuntary movement, such as LID since it was recorded in medicated patients with PD experiencing dyskinesia.^33^ In LID on‐state, we detected both theta and gamma oscillations in M1 and DLS. Recent reports describe gamma activities^8^or theta‐band oscillations^34^ in animal models of LID. In previous studies, our team only detected theta oscillations in the LID state with a low‐dose L‐dopa (6 mg/kg) used, while we investigated enhanced gamma oscillations with a higher L‐dopa dose (8 mg/kg). The main reason for the difference may be the dose of L‐dopa. Our hypothesis was supported by research on the dose effect of L‐dopa on gamma activities that high‐dose L‐dopa (12 mg/kg) could induce gamma oscillations, but low dose (7 mg/kg) failed.^8^

The electrophysiological characteristics of the M1 in the LID state are becoming a major focus of clinical research, as their alterations are the most pronounced and easy to detect, and as the M1 is a frequent target of non‐invasive stimulation. The modulation of the adaptive DBS voltage based on cortical gamma oscillatory activity as a feedback indicator provides reliable support in the clinical context.^14^ Gamma frequency‐targeted transcranial alternating current stimulation of the M1 improved the movement amplitude of subjects with PD.^35^ Our research on gamma rhythms may provide new insight for further clinical application of noninvasive stimulation and closed‐loop DBS.

In DLS, an exaggerated theta–gamma PAC was observed in our research, contrast to a previous study demonstrated enhanced theta–gamma CFC in M1 related to the on‐state dyskinesia.^8^ Discrepancy could be due to the different experiment methods and procedures. We concerned that the enhanced striatal theta–gamma PAC observed in this study is the mechanism of exaggerated broadband gamma oscillations. PAC is a potential clinical target; clinical research has revealed the enhancement of beta–gamma PAC, related closely to motor behavior overseen by the STN and motor cortex, in patients with PD,^36^ and the alleviation of cortical behavior with STN phase‐targeted stimulation to modulate cortical PAC.^14^ Moreover, beta–gamma PAC is a dependent indictor of the effect of DBS on the STN in patients with PD.^37^ Thus, theta–gamma PAC is a promising electrophysiological target for the alleviation of dyskinesia in future clinical applications. The increased theta–gamma PAC that we found may better describe the neural electrophysiological characteristics of LID and serve as a biomarker guiding closed‐loop DBS. We also detected a positive correlation between the aperiodic‐corrected beta power and aperiodic‐adjusted gamma power in the M1, suggesting that distinct mechanisms drive gamma oscillation in different regions. Excessive gamma oscillations are driven by beta oscillations in M1 while modulated by exaggerated theta–gamma PAC in DLS on‐state dyskinesia.

We observed strengthened gamma‐band coherence and PLVs after LB administration which indicated aberrant neural functional connectivity in rats with LID. Cortico‐STN coherence with a frequency‐specific topography has been detected in subjects with PD on and off of L‐dopa medication.^38^ Recent findings suggest that strengthened connectivity between the cerebellar dentate nucleus and putamen reduces dyskinetic symptoms,^39^ and functional connectivity has been used as an indicator of DBS eligibility in patients with early PD.^40^ Moreover, structural and functional connectivity predicted motor improvement independently in subjects with PD.^41^ We explore the contribution of functional connectivity to the pathology of PD and LID. What's more, the aberrant alteration of functional connectivity in the LID state found by us may provide evidence for DBS candidate selection and clinical efficacy prediction.

We demonstrated that eltoprazine, which targets 5‐HT1A/1B autoreceptors, affects gamma oscillations and thus could be applied in the treatment of LID; we explored its pharmacological profile which may be conducive to justify the optimal dosage of eltoprazine, it reduced AIMs scores, extended RTs in a dose‐dependent manner, suppressed gamma‐band activity without altering beta‐band activity, ameliorated enhanced theta–gamma PAC in DLS, and reduced M1‐DLS coherence and PLVs in gamma‐band. We also excluded the own effect of eltoprazine on LID rats without L‐dopa injection, no dyskinetic symptoms appeared in LID + E group rats. As for neural activities alteration, LID + E group rats exhibited an electrophysiological parameter resembling LID + LB + E group rats accompanied by declined gamma oscillations, theta–gamma PAC, and gamma‐band functional connectivity. In addition, neural activities in beta‐band were not influenced, all of which indicated no impact of eltoprazine alone. According to our observation during experiment procedure and data analysis, LB or eltoprazine injection alone showed no improvement during OFT on day 48 due to only induced pathological involuntary movements or lacking the anti‐parkinsonian efficacy of L‐dopa, respectively. However, eltoprazine invention combined with LB ameliorated dyskinetic symptoms with rescued locomotor activity. Drugs targeting the 5‐HT1A and/or 5‐HT1B receptors have been designed to alleviate dyskinesia. It has been proved that eltoprazine suppressed sensitization of striatonigral projections (direct pathway) and modulated striatal glutamate transmission, but had no effect on striatal ectopic DA release, which may be the basis of its anti‐dyskinetic effect.^42^ In addition, etloprazine could improve LID symptoms by recovering the long‐term potentiation and synaptic depotentiation in striatal medium spiny neurons, which was correlated to the normalization of dopamine receptor‐related cAMP/PKA and ERK/mTORC signaling pathways and the restoration of NMDA receptor subunit equilibrium.^43^ From the perspective of neuroinflammation, eltoprazine reduced the L‐DOPA‐induced upregulation of immediate‐early gene zif‐268 in striatum, delayed the onset of dyskinesia, and reserved the efficacy of L‐dopa.^44^, ^45^ Previous study demonstrated that both gamma power and gamma burst in M1 were correlated with AIMs^9^; thus, we infer that from view of in vivo electrophysiology, eltoprazine modulates exaggerated gamma activity accompanied with resumption of aberrant neural functional connectivity and information flow in M1‐DLS projection to alleviate LID symptoms. In addition, our research provided evidence that eltoprazine exerts anti‐dyskinetic effect without reducing anti‐parkinsonian efficacy of L‐dopa. We clarified the potential neural electrophysiological mechanism followed by eltoprazine invention in dyskinetic state for the first time and provide supporting evidence for its application in clinical field.

However, the findings of this study have to be seen in light of some limitations. Despite we observed excessive gamma oscillations induced by L‐dopa administration, its parameters are incompletely investigated since we only analyzed power, more characteristics and its relationship with the severity of dyskinesia are awaiting research. As we speculated that L‐dopa may affect gamma oscillations in a dose manner and a concentration gradient of L‐dopa is needed to be explored for its effects on gamma oscillations. We demonstrated that enhanced striatal theta–gamma PAC in DLS is the mechanism of exaggerated broadband gamma oscillations while excessive gamma oscillations are driven by beta oscillations in M1 in LID‐on state, more direct evidences with strong support are required for future. In addition, we confirmed the underlying electrophysiological mechanism of eltoprazine invention but the internal molecular mechanisms and synaptic plasticity alterations still lie on the table. Eltoprazine targets 5‐HT1A/1B receptors which was relative to psychiatric disorders, since the clinical manifestation included a range of non‐motor symptoms such as anxiety and depression, its effect on PD‐related psychosis needs to be explored in the future. Also, the effect of eltoprazine on PD symptoms remains to be explored both on electrophysiological and behavioral perspective.

In conclusion, this study demonstrated that L‐dopa induced exaggerated gamma oscillation in the M1 and DLS, accompanied by enhanced theta–gamma PAC in the DLS. Increased functional connectivity, represented by coherence and PLVs, was also detected based on neuroanatomical corticostriatal projections in the dyskinetic state. Eltoprazine modulated the abnormal gamma oscillation to ameliorate LID symptoms, providing compelling evidence supporting its future clinical application.

## AUTHOR CONTRIBUTIONS

YB and WZ contributed to the conception and design of the study; YB, PW, JY, ZW, HY, YD, JG contributed to the acquisition and analysis of data; YB and PW contributed to drafting the text or preparing the figures.

## CONFLICT OF INTEREST STATEMENT

The authors report no competing interests. Some figures were created with Biorender.com.

## Supporting information


Figure S1.

## Data Availability

The data that support the findings of this study are available from the corresponding author upon reasonable request.
