# Supplementary material for: Eltoprazine modulated gamma oscillations on ameliorating L‐dopa‐induced dyskinesia in rats
Source: CNS Neurosci Ther. 2023 Apr 30;29(10):2998–3013. doi: 10.1111/cns.14241 (PMC10493666; doi:10.1111/cns.14241)
Supplement: Supplementary file 1 — Figure S1. [file CNS-29-2998-s001.doc]

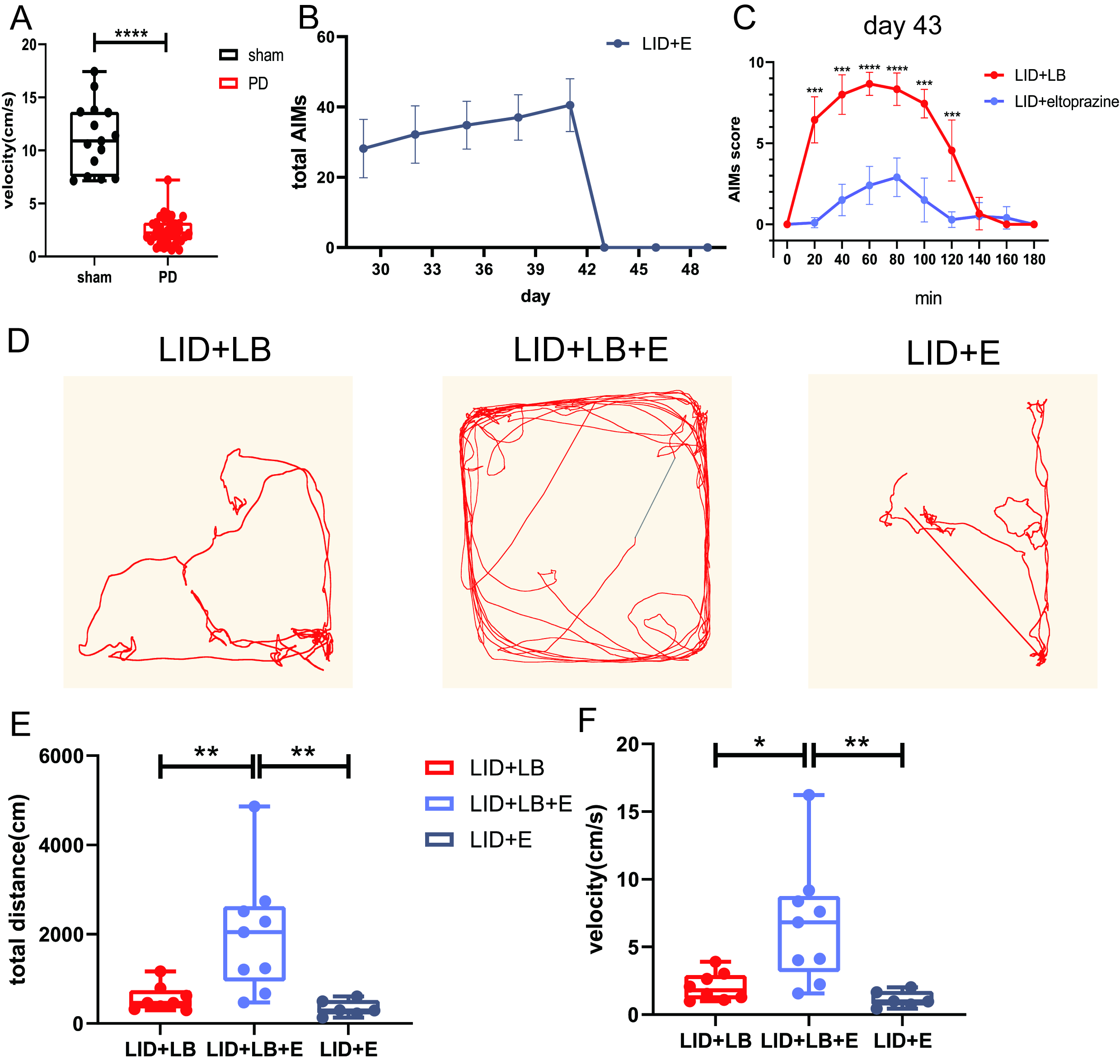


Supplementary Figure. 1 A. Velocity in the open field test. B. Total AIMs scores in LID+E group after LB or eltoprazine alone administration. C. AIMs scores after LB priming on day 43. D. Representative open field test motion trails on day 48. H, I. Comparisons of total distance traveled and velocity of LID + LB, and LID + E, LID + E groups rats. Data are means ± SEMs. **P* < 0.05, ***p* < 0.01, ****p* < 0.001, *****p* < 0.0001, Student’s t test and one-way ANOVA test followed by Tukey's multiple comparison test.
